# Congenital Leukemia: Presentation of Three Cases

**DOI:** 10.7759/cureus.95318

**Published:** 2025-10-24

**Authors:** Zuzanna Karczmarzyk, Magdalena Szuba, Joanna Krupa, Natalia Czaplińska, Bożena Kociszewska-Najman

**Affiliations:** 1 ProNeo Student Scientific Group, Department of Neonatology and Rare Diseases, Medical University of Warsaw, Warsaw, POL; 2 Department of Neonatology and Rare Diseases, Medical University of Warsaw, Warsaw, POL

**Keywords:** acute myeloid leukemia, congenital leukemia, hepatosplenomegaly in infancy, hyperleukocytosis (hl), infant, neonatal leukemia

## Abstract

Congenital leukemia is a very rare but severe disease diagnosed within the first 28 days of life. Common symptoms include hepatosplenomegaly, leukemia cutis, pallor, petechiae, leukocytosis, and thrombocytopenia. This report presents three full-term newborns diagnosed with congenital leukemia. Examination of all three patients showed hepatosplenomegaly, petechiae, and thrombocytopenia, although the symptoms appeared at a different time in each case. Due to the non-specific nature of early symptoms, cytological analysis, cytogenetic testing, and immunophenotyping are essential for establishing a definitive diagnosis. Congenital leukemia is a rare condition that requires early diagnosis, personalized treatment, and ongoing research. Despite progress in neonatal care, the prognosis of this condition remains poor, highlighting the importance of developing better diagnostic tools and therapeutic strategies.

## Introduction

Congenital leukemia (CL), also known as neonatal leukemia, is an extremely rare but aggressive condition that manifests at birth or within the first 28 days of life [1]. It occurs in 4.3 to 8.6 per million live births per year in Europe [2]. Although rare, it is the third most common neoplasm in infants, after teratoma and neuroblastoma [3].

Patients with CL most commonly present with hepatosplenomegaly, pallor, leukemia cutis (skin infiltration, often manifesting as blueberry muffin-like lesions), petechiae and neurological symptoms [3,4]. Laboratory investigation reveals the presence of immature granulocytes in the peripheral blood, hyperleukocytosis, and thrombocytopenia [3,5]. In contrast to leukemia in older children, lymphadenopathy is uncommon. In severe cases, multiple organs may be affected [3]. Due to nonspecific early symptoms, several conditions can be mistaken for CL. Cases should be differentiated from leukemoid reaction (leukocytes count of > 50 cells × 10^9^ L without the presence of neoplastic cells), which is most commonly related to congenital infection (*Cytomegalovirus*, toxoplasmosis, rubella, herpes, syphilis) or sepsis [5,6].

Therefore, cytological analysis and immunophenotyping are essential to establish a definitive diagnosis. The diagnostic criteria for CL include the following: (1) diagnosis within the first 28 days of life; (2) a significant number of primitive or blast cells in the bone marrow aspirate and elevated peripheral white blood cell counts (>25 × 10⁹/L); (3) infiltration of leukemic cells into tissues outside the blood and bone marrow; and (4) exclusion of other possible causes, such as neonatal sepsis or hemolysis [3]. Genetic abnormalities are frequently associated with CL, particularly trisomy 21 (Down syndrome), trisomy 9, trisomy 13, and Turner syndrome. Approximately 30% of neonates with Down syndrome develop transient abnormal myelopoiesis (TAM), a pre-leukemic condition that significantly increases the risk of developing acute myeloid leukemia (AML) within the first five years of life [7,8]. In addition, in utero exposure to environmental factors such as radiation, certain medications, or toxins may contribute to the pathogenesis of CL [9].

Most cases of CL are acute myelogenous leukemia, often of monocytic or monoblastic subtype. The remaining cases are typically acute lymphoblastic leukemia (ALL) of B-cell lineage [10]. The two-year survival rate for CL is approximately 25%, which is mainly due to the limited effectiveness of current treatment and the severe side effects associated with therapy [3]. In rare cases, CL can spontaneously go into remission [11].

The present study reports on three neonates diagnosed with CL. Our aim is to highlight the importance of early diagnosis and to summarize the common characteristics of CL, as early recognition is still challenging.

## Case presentation

Patient 1

Patient 1 was a male neonate born with hydrops fetalis and hematological disorders, born at 38 weeks and six days. The pregnancy was complicated by maternal nicotine use and anemia. The Apgar score was 9/9/9/9 at 1/3/5/10 minutes, respectively. The birthweight was 3370 g.

The examination revealed abdominal distension and hepatomegaly. Furthermore, the skin was pale, with petechiae and dependent edema. The symptoms appeared within the first 24 hours of life.

Laboratory tests revealed anemia, thrombocytopenia, and leukocyte lineage rejuvenation in the peripheral blood smear. The laboratory results are summarized in Table 1. TORCH infections and neonatal lupus were excluded. Echocardiographic examination revealed moderate tricuspid valve regurgitation. The magnetic resonance imaging (MRI) report revealed splenomegaly, thick periportal regions, a thick-walled gallbladder, and enlarged right axillary lymph nodes. A bone marrow biopsy was performed, including genetic analysis of the bone marrow.

**Table 1:** Complete blood count results of the first patient on day 20 and week four of life. WBC: white blood cells; LYMPH %: lymphocytes percentage; NEUT %: neutrophils percentage; MONO %: monocytes percentage; EOSYNO %: eosinophils percentage; RBC: red blood cells; HGB: hemoglobin; HCT: hematocrit; PLT: platelets.

Parameter	Day 20 of life, Result	Day 20 of life, Reference range	Week 4 of life, Result	Week 4 of life, Reference range	Units
WBC	8.24	8-24	8.65	6.8-14	10^3/µL
LYMPH %	60.2	41.2-75.4	70.6	41.2-75.4	%
NEUT %	10.6	25-35	10.7	20-25	%
MONO %	26.0	4.3-18.3	17.1	4.4-14	%
EOSYNO %	1.7	0-5	0.6	0-5	%
RBC	3.50	4.4-5.9	3.78	3.9-5.5	10^6/µL
HGB	10.4	15-19	11.4	13.5-16.5	g/dL
HCT	30.7	53-58	32.5	41-48	%
PLT	43	300-600	24	250-550	10^3/µL

At the 21st day of life, the patient was diagnosed with AML, specifically acute megakaryoblastic leukemia (AMKL) (M7). Genetic analysis of the bone marrow revealed an SRP72 mutation.

Patient 2

Patient 2 was a male neonate born by cesarean section at 38 weeks of gestation with suspected congenital hepatosplenomegaly, jaundice, and thrombocytopenia. Mother had a mild respiratory infection in the third trimester. He was assessed for 10 points in the Apgar score and weighed 3040 grams.

The physical examination showed hepatosplenomegaly, jaundice with a high level of conjugated bilirubin from the first day, and petechiae. Episodes of bradycardia were observed. Blood tests showed severe thrombocytopenia and elevated liver enzymes. The laboratory results are summarized in Table 2. The newborn received phototherapy and empirical antibiotic therapy (ampicillin and gentamicin) for seven days, until inflammatory markers normalized and blood cultures remained negative. Because of ongoing low platelet counts, the infant required seven platelet transfusions. Due to cholestasis, ursodeoxycholic acid, fat-soluble vitamins, and phenobarbital were administered.

**Table 2:** Complete blood count results of the second patient on day one, week two, and week four of life. WBC: white blood cells; LYMPH %: lymphocytes percentage; NEUT %: neutrophils percentage; MONO %: monocytes percentage; EOSYNO %: eosinophils percentage; RBC: red blood cells; HGB: hemoglobin; HCT: hematocrit; PLT: platelets; NA: indicates parameter not available; TBIL: total bilirubin; DBIL: direct bilirubin; IBIL: indirect bilirubin.

Parameter	Day 1 of life, Result	Day 1 of life, Reference range	Week 2 of life, Result	Week 2 of life, Reference range	Week 4 of life, Result	Week 4 of life, Reference range	Units
WBC	22.24	9-30	7.13	8-24	8.36	6.8-14	10^3/µL
LYMPH %	NA	18-48.6	64.9	41.2-75.4	56.8	41.2-75.4	%
NEUT %	NA	65-75	24.4	25-35	31.0	20-25	%
MONO %	NA	6.7-19.9	7.0	6.7-19.9	7.1	4.4-14	%
EOSYNO %	1.3	0-5	3.1	0-5	4.2	0-5	%
RBC	3.19	4.5-6.5	3.66	4.4-5.9	4.59	3.9-5.5	10^6/µL
HGB	12.0	16.5-23	11.3	15-19	13.6	13.5-16.5	g/dL
HCT	33.0	60-67	31.7	53-58	37.2	41-48	%
PLT	103	300-600	61	300-600	100	250-550	10^3/µL
TBIL	15.11	0.15-3	8.79	0.15-1	4.98	0.15-1	mg/dL
DBIL	4.36	0-0.6	7.97	0-0.6	4.85	0-0.6	mg/dL
IBIL	10.75	NA	0.82	NA	0.13	0.2-1	mg/dL

Additional tests excluded hereditary spherocytosis, hemophagocytic syndrome, red blood cell enzyme defects, and galactosemia. After bone marrow aspiration and trephine biopsy on the 26th day of life, a diagnosis of AMKL was made.

Whole-exome sequencing of blast cells revealed four autosomal trisomies involving chromosomes 14, 15, 19, and 21, but no disease-causing single-nucleotide variants were identified. The infant was transferred to the pediatric hematology department for further specialized treatment.

Patient 3

A full-term female newborn was admitted to the hospital on the 14th day of life for evaluation and treatment of thrombocytopenia. She was born at 40 weeks of gestation, with a birthweight of 2800 grams and 10 points in the Apgar score. The pregnancy was complicated by gestational diabetes mellitus (GDM, type 2) and a viral infection during the second trimester. On the 10th day postpartum, the mother tested positive for COVID-19. She is also a carrier of the factor V Leiden mutation.

**Figure 1:**
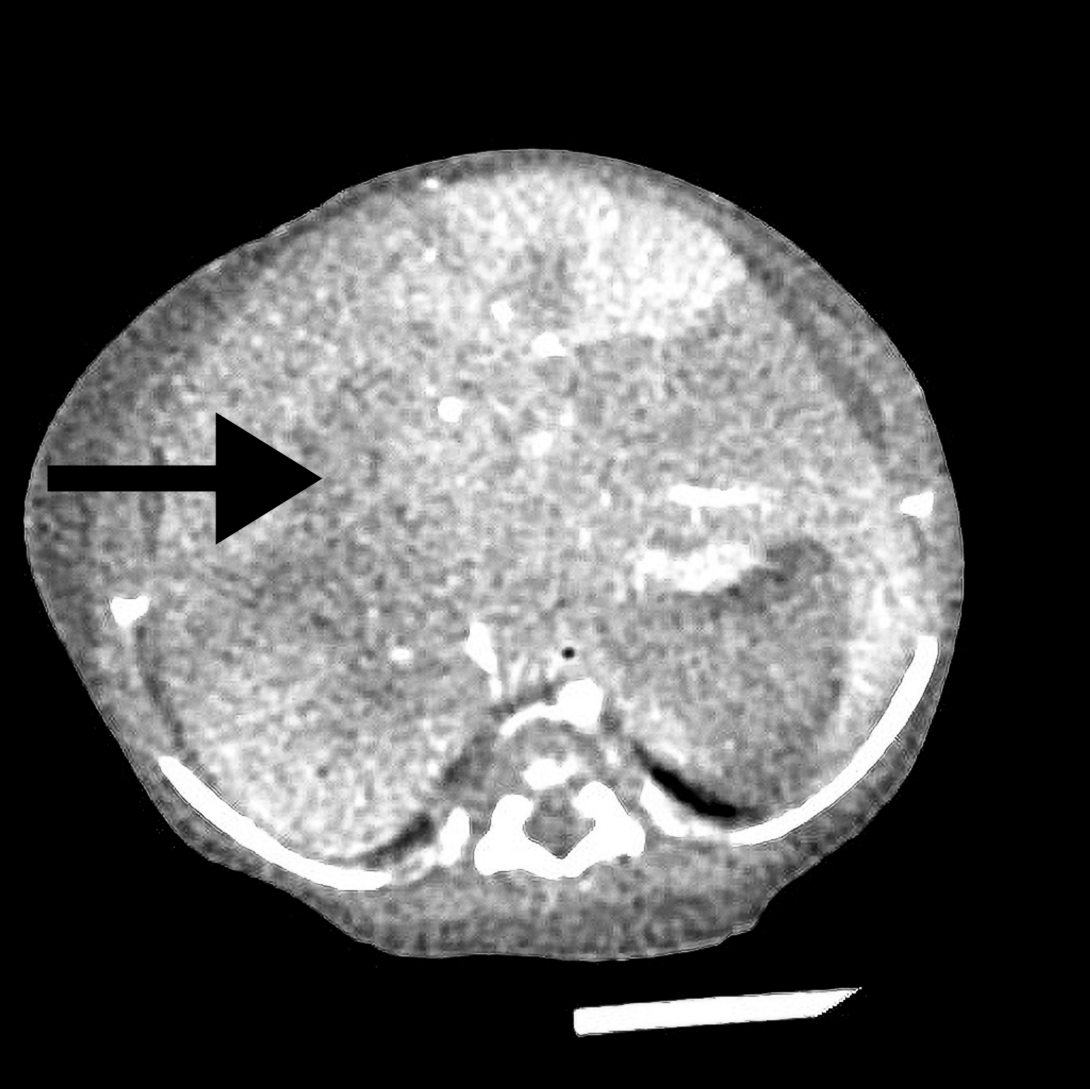
Abdominal CT of the third patient. CT scan showing hepatomegaly. The arrow points to the area of interest.

The infant had hepatosplenomegaly and signs of an upper respiratory tract infection. Blood test results showed pancytopenia, hyperferritinemia, hypofibrinogenemia, and elevated soluble CD25, raising a strong suspicion of hemophagocytic lymphohistiocytosis (HLH). The laboratory results are summarized in Table 3. The computed tomography revealed hepatomegaly with heterogeneous liver parenchyma (Figure 1).

**Table 3:** Complete blood count results of the third patient at week two of life. WBC: white blood cells; LYMPH %: lymphocytes percentage; NEUT %: neutrophils percentage; MONO %: monocytes percentage; EOSYNO %: eosinophils percentage; RBC: red blood cells; HGB: hemoglobin; HCT: hematocrit; PLT: platelets.

Parameter	Week 2 of life, Result	Week 2 of life, Reference range	Units
WBC	11.74	8-24	10^3/µL
LYMPH %	42.5	41.2-75.4	%
NEUT %	17.5	25-35	%
MONO %	35.2	5.2-20.6	%
EOSYNO %	1.8	0-5	%
RBC	3.15	4.4-5.9	10^6/µL
HGB	10.1	15-19	g/dL
HCT	28.9	53-58	%
PLT	34	300-600	10^3/µL

Treatment was started according to the HLH-2004 protocol with dexamethasone, tocilizumab, intravenous immunoglobulin (IVIG), and etoposide. However, the infant developed severe neutropenia and did not improve, so treatment was stopped. On the 17th day of life, a bone marrow biopsy was performed. Congenital HLH was not confirmed. Peripheral blood smear showed a marked left shift with 19% nucleated red blood cells. A follow-up bone marrow aspirate revealed numerous blasts consistent with megakaryoblasts. Immunophenotyping showed 53% of cells with low CD45 expression (about 30% of all bone marrow cells). On the 38th day of life, a diagnosis of AMKL (subtype M7) was made, and treatment was started according to the AML-BFM 2017 protocol.

After diagnosis, the infant experienced episodes of desaturation, weight loss, peripheral edema, hypoalbuminemia, hepatomegaly, and tachycardia. Her condition worsened, and due to metabolic acidosis and desaturation, she was transferred to the intensive care unit. Sepsis due to *Stenotrophomonas maltophilia* was diagnosed. Despite intensive treatment, she developed pneumothorax, severe anemia, oliguria, petechiae, liver failure, pulmonary bleeding, hypotension, and bradycardia. The infant died shortly afterward.

A comparative summary of three patients' characteristics, including diagnosis, symptom onset, complete blood count results, hepatomegaly, and day of confirmed diagnosis, is presented in Table 4.

**Table 4:** Comparison of clinical features of the three patients. Comparative overview of clinical features of patients 1–3.

Feature	Patient 1	Patient 2	Patient 3
Diagnosis	Acute megakaryoblastic leukemia	Acute megakaryoblastic leukemia	Acute megakaryoblastic leukemia
Symptom onset	Within the first 24 hours of life	Prenatally	14^th^ day of life
Complete blood count	Anemia, thrombocytopenia, and leukocyte lineage rejuvenation in the peripheral blood smear	Anemia, thrombocytopenia, leukocytosis	Pancytopenia
Hepatomegaly	Present	Present	Present
Confirmed diagnosis day	21st day of life	26th day of life	38th day of life

## Discussion

Congenital acute leukemia is very rare. According to the study from the Northern Health Region of England, it occurs in 8.6 per million live births per year. In infants within four weeks, the value would decrease by more than half [12]. AMKL accounts for approximately 4-15% of pediatric AML cases [13].

Patients with AML may present with fatigue, easy bruising, excessive bleeding, and shortness of breath. Physical examination reveals pallor of the skin and hepatosplenomegaly. Lymphadenopathy is not common. Laboratory tests reveal anemia, thrombocytopenia, and leukocytosis. In our case series, all patients presented with anemia and thrombocytopenia. However, the third patient revealed pancytopenia. Serious complications such as disseminated intravascular coagulation (DIC) can be observed. It may be associated with oral mucosal hemorrhages, petechiae, and bleeding from intravenous insertion sites [14]. In our case, all three patients presented with hepatosplenomegaly, petechiae, and thrombocytopenia. However, the onset of symptoms occurred at a different time in each patient. The symptoms were observed prenatally only in the second case, and in the other two cases on the 1st and 14th day of life, respectively. Of these three cases, all patients have been diagnosed after three weeks.

One of the diagnosis challenges is to distinguish congenital myeloid leukemia from a leukemoid reaction, caused by infection, hemolysis, or severe asphyxia, and transient myeloproliferative disorder (TMD). Firstly, symptoms are very similar; however, TMD resolves spontaneously [15].

A common feature in our three patients was hepatosplenomegaly. However, its occurrence does not always indicate a malignant disease. In neonates, hepatosplenomegaly can be associated with congenital infections such as cytomegalovirus (CMV) infection or toxoplasmosis, as well as with metabolic disorders. In CMV infection, thrombocytopenia and elevated liver enzymes are typical findings. However, normal blood counts at the initial stage do not exclude the presence of infection.

Diagnostic evaluation of AML includes complete blood count, cytochemistry, immunophenotyping, karyotyping, fluorescence in situ hybridization, and molecular diagnostics of the bone marrow [16]. The French-American-British (FAB) classification system categorizes AML into subtypes based on morphological and cytochemical features. It considers lineage-associated phenotypes, including undifferentiated, myeloid, monoblastic, erythroblastic, or megakaryoblastic [17]. However, the diagnosis of AMKL needs to be confirmed by immunophenotyping. AMKL is often associated with bone marrow fibrosis, which may result in a dry tap during aspiration and consequently lead to an underestimation of the blast percentage. That is why in cases where the blast count is less than 20% the bone marrow biopsy has to be repeated [16].

CL is often associated with genetic abnormalities, particularly trisomy 21 (Down syndrome), trisomy 9, and trisomy 13 [18]. Children with Down syndrome (DS) or mosaic DS have a 14- to 20-fold increased risk of developing acute leukemia [19]. AMKL accounts for approximately 50% of the AML in children with Down syndrome [20]. However, in our cases, none of the patients had Down syndrome. The second patient's examination revealed trisomy 21 in blast cells, but the karyotype of the patient was normal.

## Conclusions

Congenital acute leukemia is a rare and often fatal condition that poses significant diagnostic and therapeutic challenges in neonates. Despite its infrequent occurrence, it should be considered during differential diagnosis. Prompt diagnosis and initiation of appropriate treatment are essential. Despite common features such as hepatosplenomegaly, petechiae, and thrombocytopenia, the timing of symptom onset and disease progression varied substantially among the patients. Bone marrow examination remains essential, particularly when peripheral blood findings are inconclusive. Genetic studies may provide further diagnostic clarity. Genetic testing revealed an SRP72 mutation in one patient and complex chromosomal aberrations (trisomies 14, 15, 19, and 21) in another, suggesting the presence of distinct molecular mechanisms leading to a similar clinical phenotype. In one case, environmental and infectious factors may have contributed, indicating that early hematologic manifestations can result from both genetic and immunologic disturbances.

Despite advances in neonatal care and treatment protocols, the prognosis of congenital AML remains poor. These reports emphasize the importance of continued research into the pathogenesis, early detection, and tailored treatment strategies for congenital leukemia in neonates.
